# When tradition meets technology: perceived value integration and consumer purchase behavior in the smart agriculture era

**DOI:** 10.3389/fnut.2026.1834423

**Published:** 2026-05-28

**Authors:** Yan Zheng, Ziyi Liu, Dayu Cao, Xiaoying Yao, Geng Liu, Qi Tian

**Affiliations:** 1 School of Economics and Management, Jiangxi Agricultural University, Nanchang, China; 2 Vocational Normal College, Jiangxi Agricultural University, Nanchang, China

**Keywords:** perceived value integration, purchase behavior, response surface analysis, technological trust, traditional farming culture

## Abstract

**Introduction:**

Smart agriculture prompts consumers to reconcile recognition of traditional farming culture with trust in smart agricultural technology when evaluating agri-food products. This study examines how the congruence between these two perceptions influences perceived value integration and purchase behavior.

**Methods:**

Survey data from 703 Chinese consumers were analyzed using structural equation modeling and response surface analysis. The study assessed the congruence and incongruence effects of recognition of traditional farming culture and trust in smart agricultural technology on perceived value integration, as well as the mediating role of perceived value integration and the moderating role of trust in government.

**Results:**

Greater congruence between recognition of traditional farming culture and trust in smart agricultural technology was associated with higher perceived value integration, whereas incongruence reduced it regardless of direction. Perceived value integration mediated the relationship between the congruence of these two perceptions and purchase behavior. Trust in government negatively moderated the effect of this congruence on perceived value integration.

**Discussion:**

The alignment of cultural recognition and technological trust plays an important role in shaping consumer responses to smart agriculture. Cultural narratives and technological credibility should be integrated into marketing communication, while institutional endorsement should be used cautiously to avoid weakening consumers’ independent value judgment.

## 1 Introduction

Agriculture is undergoing a profound technological transformation. Smart agriculture, supported by the Internet of Things, big data, and artificial intelligence, is reshaping how food is produced, processed, and distributed (1–3). These innovations are widely recognized as essential pathways to the challenges of food safety, sustainability, and production efficiency (3, 4). Nevertheless, despite rapid technological progress, consumer acceptance of smart agricultural products in China still lags behind industrial development (5). In recent years, a growing number of agricultural products featuring digital traceability, intelligent monitoring, blockchain certification, and AI-assisted production management have entered the Chinese market (6). However, their market performance has not always met technological expectations. Many consumers remain cautious about whether such products are genuinely safer, more nutritious, or more trustworthy than conventional alternatives (7, 8). In practice, some products promoted with labels such as “smart,” “digital,” or “traceable” have failed to gain stable consumer recognition, partly because consumers are unfamiliar with the underlying technologies and partly because technological claims do not automatically translate into perceived value at the point of purchase (9). This gap is especially salient in China, where food consumption is deeply embedded in traditional agricultural culture and consumers often attach considerable importance to authenticity, naturalness, and familiarity when evaluating agri-food products (10). As a result, smart agricultural products may face a dual legitimacy challenge: they must not only demonstrate technological credibility but also resonate with culturally rooted expectations about farming and food. This practical dilemma suggests that the low acceptance of smart agricultural products cannot be fully explained by technology-related concerns alone and that a deeper culture–technology perspective is needed.

Previous studies have interpreted this hesitation primarily through the technology acceptance model and risk perception theory, emphasizing issues such as technological unfamiliarity, safety concerns, or perceived uncertainty (11–13). Although these frameworks have yielded valuable insights, they focus mainly on rational or risk-based evaluations and often overlook deeper cultural dimensions embedded in food-related decisions. In societies with enduring agricultural traditions, food consumption is not merely functional but also a cultural practice intertwined with collective memory, inherited identity, and emotional attachment (14–16). Consequently, the adoption of smart agricultural products goes beyond technological assessment and demands a reconciliation between agricultural values rooted in tradition and those oriented toward technological futures (14).

This reconciliation constitutes a process of value negotiation, in which emotional and social values derived from traditional farming culture converge with—or conflict against—the functional and cognitive values introduced by advanced agricultural technologies (17, 18). Existing studies have tended to examine either cultural heritage (19, 20) or technology trust (21, 22) as isolated predictors of consumer behavior, while comparatively limited attention has been devoted to their interactive effects. The joint influence of these dual value orientations—and their integration in consumer cognition—remains underexplored. Understanding this integration is essential for capturing the complexity of decision-making in the smart agriculture context.

To address this conceptual void, this study introduces perceived value integration (PVI) as a central mediating process. Drawing on the theory of consumption values (18), PVI reflects the extent to which consumers merge multiple value dimensions—functional, emotional, cognitive, and social—into a coherent evaluation. Purchasing decisions typically stem from a synthesis of value inputs rather than a single factor, and the perceived harmony among them is decisive. To measure this mechanism, response surface analysis (RSA) is employed to investigate how congruence between recognition of traditional farming culture (RTFC) and trust in smart agricultural technology (TSAT) affects PVI in a nonlinear pattern. This approach reveals that high–high cultural-technological congruence enhances value coherence, whereas mismatched configurations (e.g., high RTFC–low TSAT or high TSAT–low RTFC) generate cognitive dissonance and subsequently weaken value integration.

Beyond individual cognition, consumer evaluations of agricultural innovations are shaped by institutional trust. According to trust theory (23), trust in government (TG) provides an external assurance mechanism that influences how individuals interpret technology-mediated agricultural systems (24). Notably, this study demonstrates that overly strong institutional trust can weaken the constructive role of culture-technology alignment on PVI, as excessive reliance on governmental endorsement may reduce consumers’ motivation to conduct autonomous, value-based assessments.

Accordingly, this study seeks to address three research questions:

1. How does the congruence or incongruence between RTFC and TSAT influence PVI in a nonlinear manner?
2. Does PVI mediate the linkage between cultural-technological congruence and consumer purchase behavior?
3. How does TG moderate the relationship between cultural-technological congruence, PVI, and subsequent purchase behavior?

By integrating consumption value theory with trust theory, this study contributes to the emerging literature on consumer behavior in the smart agriculture era. Theoretically, it conceptualizes culture-technology fit as a novel explanatory construct describing how consumers reconcile traditional cultural values with technological trust when forming purchase decisions. By identifying PVI as a key psychological mechanism and employing RSA to uncover nonlinear effects of cultural-technological alignment, this research deepens the theoretical understanding of consumer cognition in hybrid cultural-technological contexts. In practical terms, the findings offer guidance for aligning cultural resonance with technological credibility. Firms should embed cultural authenticity into technological narratives through brand storytelling and experiential design, while policymakers should calibrate institutional endorsement to preserve consumer agency and value-based judgment. Collectively, these insights offer both a theoretical foundation and a practical pathway for fostering consumer-centered sustainable smart agriculture.

## 2 Literature review and research hypotheses

### 2.1 Smart agricultural products, culture-technology fit, and consumer purchase behavior

Smart agricultural products refer to agri-food products whose production, processing, quality control, or circulation is supported by digital and intelligent technologies such as the Internet of Things, sensors, big data analytics, artificial intelligence, and blockchain-based traceability systems (25). Compared with conventional agricultural products, smart agricultural products are characterized not only by the technological embeddedness of the production process but also by greater visibility, traceability, and data-driven quality assurance throughout the supply chain (26). In the consumer domain, however, acceptance of such products is not determined solely by objective technological performance (21). Rather, purchase-related responses are shaped by whether consumers perceive these products as both technologically credible and culturally meaningful (27).

In this study, consumer purchase behavior refers to the evaluative and decision-making responses through which consumers develop purchase tendencies toward smart agricultural products after assessing their overall value, credibility, and relevance to personal needs. Prior studies have shown that purchase-related behavior in food contexts is jointly influenced by quality perceptions, safety beliefs, emotional attachment, symbolic meaning, and institutional assurance (28, 29). In culturally sensitive consumption domains such as food and agriculture, buying decisions often reflect not only utilitarian calculations but also identity expression, emotional resonance, and trust formation (30, 31).

To explain this complexity, the present study introduces culture–technology fit (CTF) as a core explanatory construct. CTF is defined as the degree of congruence between recognition of traditional farming culture (RTFC) and trust in smart agricultural technology (TSAT). RTFC captures consumers’ recognition of the symbolic, emotional, and identity-related meanings embedded in traditional farming culture, whereas TSAT reflects confidence in the reliability, safety, and effectiveness of smart agricultural technologies. In this study, CTF is operationalized as the congruence or matching pattern between RTFC and TSAT and is examined using response surface analysis. According to fit theory, consistency across important evaluative dimensions tends to generate psychological harmony and more favorable outcomes (32). Accordingly, when RTFC and TSAT are aligned, consumers are more likely to form coherent and favorable evaluations of smart agricultural products; when they are misaligned, consumers may experience evaluative conflict.

### 2.2 Perceived value integration: from trade-offs to synergy

The theory of consumption values provides a multidimensional lens for understanding consumer decision-making, positing that choices are shaped by the interaction among functional, emotional, social, epistemic, and conditional values (18). In food-related contexts, this framework is particularly relevant, as purchasing decisions often extend beyond utilitarian considerations such as safety and quality (functional value) to include social belonging (social value) and emotional attachment to heritage and land (emotional value) (33, 34).

Traditional applications of this framework often treat value dimensions as competitive or mutually substitutable, implying that consumers must trade off emotional priorities against functional concerns (35, 36). However, in the context of smart agriculture, such dichotomous reasoning is increasingly inadequate. Consumers often seek value coherence, namely, an integrative experience that combines the symbolic meanings of traditional farming culture with the functional and epistemic benefits derived from agricultural technologies (14). To capture this process, this study introduces perceived value integration (PVI), defined as the degree to which consumers reconcile and harmonize cultural and technological values into a unified evaluative schema (37). High PVI indicates that consumers perceive traditional authenticity and technological advancement as compatible and mutually reinforcing, whereas low PVI reflects fragmentation in value perception.

### 2.3 Cultural recognition, technological trust, and governmental trust in smart agriculture

Trust is a pivotal mechanism for reducing uncertainty and perceived risk in food consumption (38, 39). In the context of smart agriculture, consumer evaluation is influenced not only by technological trust and institutional trust but also by culturally grounded assurance derived from recognition of traditional farming culture.

Traditional farming culture evokes authenticity, purity, ecological balance, and social continuity, thereby generating emotional comfort and symbolic identification (40). Such culturally grounded assurance may provide consumers with a familiar and meaningful interpretive framework when evaluating smart agricultural products. By contrast, technological trust reflects confidence in the reliability, transparency, and safety of digital agricultural systems, including sensor-based monitoring, algorithmic decision support, and traceability mechanisms (41, 42). Institutional trust, operationalized in this study as trust in government (TG), provides external assurance through regulation, certification, and public governance (24, 43).

According to trust transfer theory, institutional trust may shape how consumers process and rely on other evaluative cues (44, 45). When governmental endorsement is highly salient, consumers may rely more on external institutional assurance than on their own integration of cultural and technological evaluations (46). Under such conditions, the effect of culture–technology fit on value integration may be weakened because institutional trust can partially substitute for consumers’ autonomous assessment of whether tradition and technology fit together. By contrast, when TG is relatively low, consumers may rely more heavily on their own judgment regarding the alignment between RTFC and TSAT, making CTF a more decisive driver of PVI. In addition, institutional trust may increase consumers’ confidence in acting on their integrated value judgments. This reasoning provides the theoretical basis for examining the moderating role of TG in the proposed model.

### 2.4 RTFC–TSAT matching and PVI: a nonlinear effect

According to the theory of consumption values (18), consumer evaluations of agricultural products extend beyond utilitarian attributes such as safety, quality, and functionality to include symbolic meanings embedded in cultural identity and emotional attachment. In the context of smart agriculture, RTFC and TSAT are not substitutes; rather, they jointly shape consumers’ holistic perceptions of value (14, 47). When consumers cognitively and emotionally integrate traditional values with technological values, they are more likely to develop a stronger sense of PVI.

Drawing on fit theory (32), this study conceptualizes the joint effect of RTFC and TSAT as a form of culture–technology fit. From a congruence perspective, alignment between recognition of traditional farming culture and trust in smart agricultural technology should promote PVI because such alignment creates evaluative harmony. In particular, when both RTFC and TSAT are high, cultural authenticity and technological assurance can reinforce each other, generating synergistic perceptions of value. By contrast, when RTFC and TSAT are incongruent, consumers are more likely to experience cognitive dissonance and value fragmentation, which undermines their ability to integrate cultural and technological values into a coherent evaluation.

H1a: When RTFC and TSAT are both high and congruent (i.e., a high RTFC–high TSAT match), PVI will be significantly enhanced.

H1b: When RTFC and TSAT are highly incongruent (i.e., high RTFC–low TSAT or low RTFC–high TSAT), PVI will significantly decrease.

### 2.5 Mediating effect of PVI

Consumers’ purchase behavior regarding smart agricultural products is rarely determined by a single cognitive or affective factor. Rather, it depends on how multiple value dimensions are synthesized into an overall perception of worth (18, 34, 48). PVI captures this synthesis process by reflecting the extent to which consumers combine functional, emotional, and symbolic values into a coherent evaluation (37).

When RTFC and TSAT are aligned, the integration of cultural reassurance and technological reliability increases consumers’ confidence and reinforces the perceived meaningfulness of the product, thereby promoting purchase-related behavior. In contrast, incongruence produces evaluative strain, resulting in fragmented value judgments and a lower likelihood of purchase. Therefore, PVI is posited as the central psychological mechanism through which the congruence or incongruence between RTFC and TSAT influences consumer purchase behavior.

H2: PVI mediates the relationship between RTFC-TSAT matching and purchase behavior (PB).

### 2.6 Moderating effect of trust in government

In the context of agri-food consumption, trust in government (TG) represents an important form of institutional trust that shapes how consumers interpret and evaluate information related to smart agricultural products (43). Government regulation, certification mechanisms, and institutional safeguards can reduce consumers’ perceived risk and strengthen their confidence in product quality, safety, and market orderliness (24).

According to trust transfer theory, TG, as an external institutional assurance cue, can reinforce consumers’ reliance on other positive evaluative cues (44, 45). When consumers have a high level of trust in government, they are more likely to regard the alignment between recognition of traditional farming culture and trust in smart agricultural technology as a credible and valuable product signal, thereby strengthening the positive effect of RTFC–TSAT matching on perceived value integration (PVI). At the same time, a higher level of TG can also enhance consumers’ confidence in acting on their integrated value judgments, thereby strengthening the positive effect of PVI on purchase behavior.

H3a: TG positively moderates the relationship between RTFC–TSAT matching and PVI, such that the positive effect of RTFC–TSAT matching on PVI is stronger when TG is high.

H3b: TG positively moderates the relationship between PVI and purchase behavior, such that the positive effect of PVI on purchase behavior is stronger when TG is high.

### 2.7 Integrated research framework

**Figure 1:**
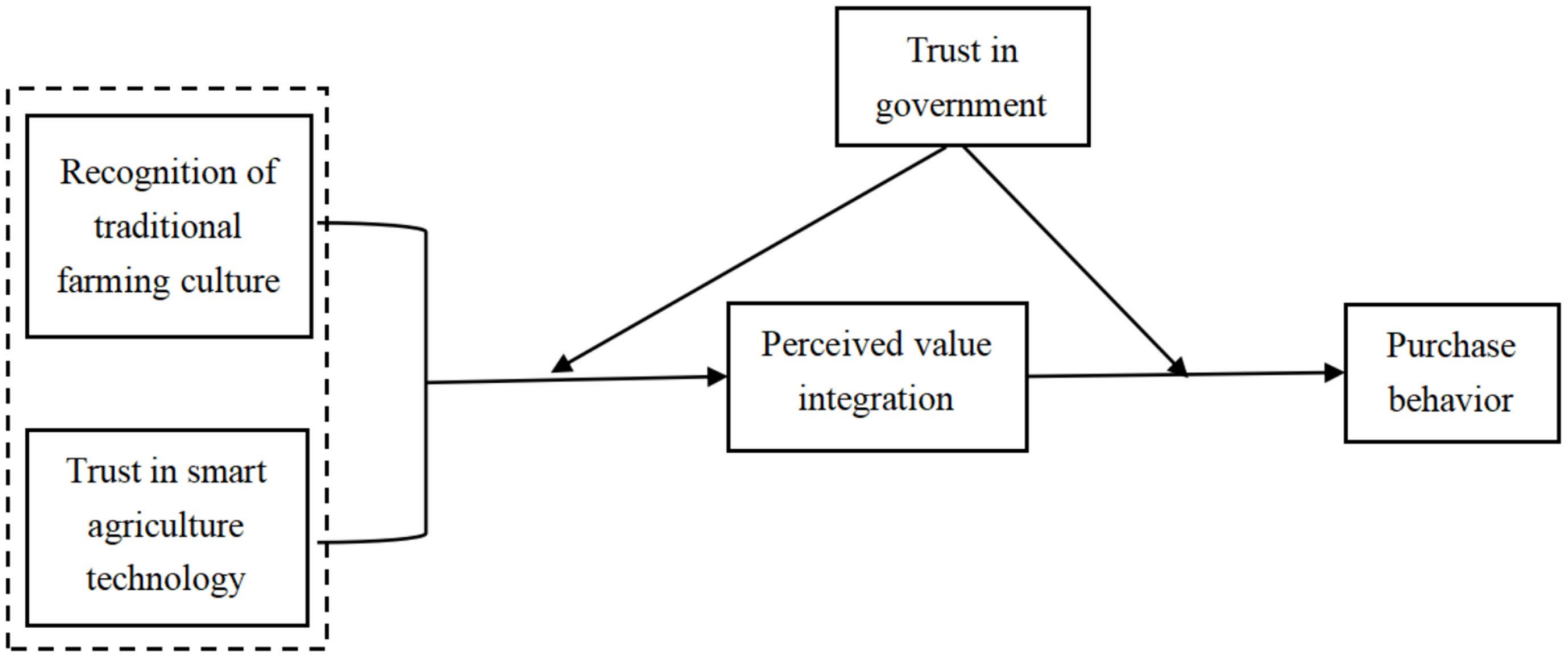
The conceptual model.

By synthesizing insights from consumption value theory, fit theory, and trust transfer theory, this study develops an integrated framework to explain consumer purchase behavior toward smart agricultural products. Methodologically, this study combines response surface analysis (RSA) and structural equation modeling (SEM). RSA is employed to examine the congruence and incongruence effects of RTFC and TSAT on PVI, whereas SEM is used to test the mediating role of PVI, the moderating effect of TG, and the overall structural relationships among the focal constructs. The conceptual model is presented in Figure 1.

## 3 Methodology

### 3.1 Data collection and sample profile

This study employed a structured questionnaire survey to examine consumer perceptions and purchasing behavior toward smart agricultural products in China. In this study, smart agricultural products are defined as agri-food products whose production, quality control, traceability, or distribution is supported by digital or intelligent technologies, such as Internet of Things (IoT)-based monitoring, sensor systems, AI-assisted decision-making, big-data management, and blockchain-enabled traceability. To ensure a relatively consistent understanding of this concept among respondents, a brief definition and several illustrative examples were provided at the beginning of the questionnaire, including products marketed with features such as intelligent traceability, digital quality monitoring, and smart production management.

Data were collected between July and August 2025 through both online and offline channels. The online survey was administered via Credamo,^1^ a widely used professional survey platform in China that provides access to respondents from diverse geographic backgrounds. The offline survey was conducted by trained members of the research team in selected urban commercial areas, community spaces, and agricultural product retail outlets, with the aim of including consumers with varying levels of digital access and purchasing experience.

A stratified quota sampling approach was adopted with reference to regional population distributions reported in the China Statistical Yearbook 2024 (National Bureau of Statistics).^2^ To enhance geographic diversity, the sample included selected provinces and municipalities from three major regions characterized by different levels of economic and technological development: eastern China (Beijing, Shanghai, Jiangsu, Zhejiang, Shandong, and Guangdong), central China (Hunan, Hubei, Henan, and Jiangxi), and western China (Sichuan, Shaanxi, and Chongqing). Quotas were assigned broadly in proportion to the population size of these three macro-regions, while also considering practical accessibility and the need to capture regional differences in exposure to smart agriculture.

During data collection, the research team first established target quotas for each region and then monitored the distribution of returned questionnaires to prevent excessive concentration in any single province or city. Within each region, efforts were made to include respondents with diverse demographic characteristics, particularly in terms of gender, age, and income, provided that they had experience purchasing agricultural or food products for themselves or their households. This sampling design improved the relevance of the sample for addressing the research objectives.

Prior to the formal survey, a pilot test involving 30 respondents was conducted to assess item clarity, wording, and conceptual comprehensibility. Minor revisions were subsequently made to improve readability and reduce ambiguity. The final questionnaire consisted of two sections: (1) demographic information and (2) measurement items for five focal constructs (RTFC, TSAT, PVI, TG, and PB). All items were measured using a five-point Likert scale ranging from 1 (“strongly disagree”) to 5 (“strongly agree”).

A total of 875 questionnaires were collected. After excluding responses with substantial missing data, evident response patterns, and failures on basic quality checks, 703 valid questionnaires were retained for analysis, yielding an effective response rate of 80.3%. The demographic characteristics of the sample are reported in Table 1, which indicates a relatively balanced sample structure. As noted by Dong et al. (49), younger and better-educated consumers tend to be more receptive to innovative agricultural products. In this regard, the composition of the final sample is broadly consistent with the consumer segment most likely to engage with smart agricultural products.

**Table 1:** Sample characteristics (*N* = 703).

Category	Group	*n*	%
Gender	Male	378	53.8
	Female	325	46.2
Age	18–30 years old	289	41.1
	31–40 years old	310	44.1
	41–50 years old	87	12.4
	50 years old and above	17	2.4
Monthly income (RMB)	Less than 3,000	17	2.4
	3,000–5,000	90	12.8
	5,001–8,000	200	28.4
	8,001–12,000	224	31.9
	More than 12,000	172	24.5
Education	Junior high school or below	4	0.6
	High school or technical secondary school	23	3.3
	Junior college or undergraduate	557	79.2
	Postgraduate and above	119	16.9

### 3.2 Measures

All constructs were measured using established multi-item scales from prior literature, with minor wording adjustments to improve contextual relevance for the smart agriculture setting. All items were scored on a five-point Likert scale ranging from 1 (“Strongly disagree”) to 5 (“Strongly agree”); see Appendix for the complete questionnaire. Recognition of traditional farming culture (RTFC) was operationalized through four items derived from Jayasekara (50) and Tanko (51). Trust in smart agricultural technology (TSAT) was assessed using four items adapted from Alexander et al. (41), Leschanowsky et al. (52), and Omrani et al. (53). Perceived value integration (PVI) was measured with three items sourced from Yang et al. (37) and Zscheischler et al. (54). Trust in government (TG) was captured through four items from Chang et al. (24) and Tian et al. (43). Purchase behavior (PB) was assessed with three items based on Stranieri et al. (55).

### 3.3 Analytical methods

Data analysis proceeded using AMOS 24.0 and SPSS 29.0 following a four-stage procedure. First, confirmatory factor analysis (CFA) was carried out to verify the reliability and validity of the measurement model, including Cronbach’s alpha, composite reliability (CR), and average variance extracted (AVE) to establish convergent and discriminant validity. Second, response surface analysis (RSA) was employed to assess the nuanced effects of congruence and incongruence between RTFC and TSAT on PVI. This technique offers a more refined analytical lens than traditional methods by capturing how alignment (or misalignment) jointly shapes integrated value perceptions. Third, polynomial regression was used to test the mediating role of PVI in the link between RTFC–TSAT congruence and PB, following the methodological guidance of Edwards and colleagues (32, 56). Finally, the moderating effect of TG was examined using Hayes’s PROCESS macro (Model 58) in SPSS 29.0 (57) to determine the extent to which TG influences the strength of the proposed relationships.

## 4 Results

### 4.1 Common method bias

Given that all variables were measured using a single self-reported survey, the potential for common method bias (CMB) was addressed using both procedural and statistical safeguards in line with established guidelines (58, 59). Procedurally, clear and neutral wording was used throughout the questionnaire to avoid ambiguity, anonymity was guaranteed to reduce evaluation apprehension, and items were grouped by construct using varied formats to introduce psychological separation between predictor and outcome variables.

Statistically, Harman’s single-factor test was conducted through exploratory factor analysis, and the first factor accounted for only 32.9% of the total variance, well below the 50% threshold for severe bias. A confirmatory single-factor model was also tested by loading all 18 items onto one factor, which yielded poor model fit (*χ*^2^ = 2,924.96, df = 135, GFI = 0.627, AGFI = 0.527, CFI = 0.512, TLI = 0.446, RMSEA = 0.172) (Table 2). A chi-square difference test further confirmed that the hypothesized five-factor model provided a significantly superior fit compared with the single-factor solution (Δ*χ*^2^ (10) = 2,647.462, *p* < 0.001).

**Table 2:** Measurement model statistics.

Models	Variable	*χ*^2^ (df)	GFI	AGFI	CFI	TLI	RMSEA	Δ*χ*^2^ (df)
Five-factor model	RTFC, TSAT, PVI, TG, PB	277.498 (125)	0.959	0.944	0.973	0.967	0.042	–
Four-factor model	RTFC + TSAT, PVI, TG, PB	1,017.08 (129)	0.808	0.745	0.845	0.816	0.099	739.582 (4)^***^
Three-factor model	RTFC + TSAT + PVI, TG, PB	1,605.464 (132)	0.752	0.679	0.742	0.701	0.126	1,327.966 (7)^***^
Two-factor model	RTFC + TSAT + PVI + TG, PB	2,236.772 (134)	0.685	0.598	0.632	0.580	0.150	1,959.274 (9)^***^
One-factor model	RTFC + TSAT + PVI + TG + PB	2,924.96 (135)	0.627	0.527	0.512	0.446	0.172	2,647.462 (10)^***^

***p < 0.001; *N* = 703.

Together, these results indicate that common method bias is unlikely to compromise the interpretation of findings.

### 4.2 Reliability and validity

The reliability and validity of the measurement model were evaluated using CFA. As reported in Table 3, all five constructs exhibited high internal consistency, with Cronbach’s alpha and CR values exceeding the recommended threshold of 0.70 (60). Convergent validity was established, as the AVE for each construct surpassed the 0.50 benchmark (61). Discriminant validity was also confirmed using the Fornell–Larcker criterion: the square root of each AVE (diagonal, bolded) was greater than its correlations with the other constructs. These indicators collectively affirm the robustness of the measurement model.

**Table 3:** The correlation coefficients of the variables.

Variable	Cronbach’s alpha	CR	AVE	RTFC	TSAT	PVI	TG	PB
RTFC	0.803	0.804	0.508	**0.713**				
TSAT	0.835	0.835	0.559	0.322^**^	**0.748**			
PVI	0.816	0.817	0.598	0.296^**^	0.196^**^	**0.773**		
TG	0.846	0.846	0.579	0.411^**^	0.327^**^	0.205^**^	**0.761**	
PB	0.894	0.896	0.742	0.499^**^	0.370^**^	0.335^**^	0.313^**^	**0.861**

**p < 0.01; *N* = 703. The square root of AVE for discriminant validity is illustrated in bold font.

### 4.3 Hypothesis testing

#### 4.3.1 Testing the structural model

**Figure 2:**
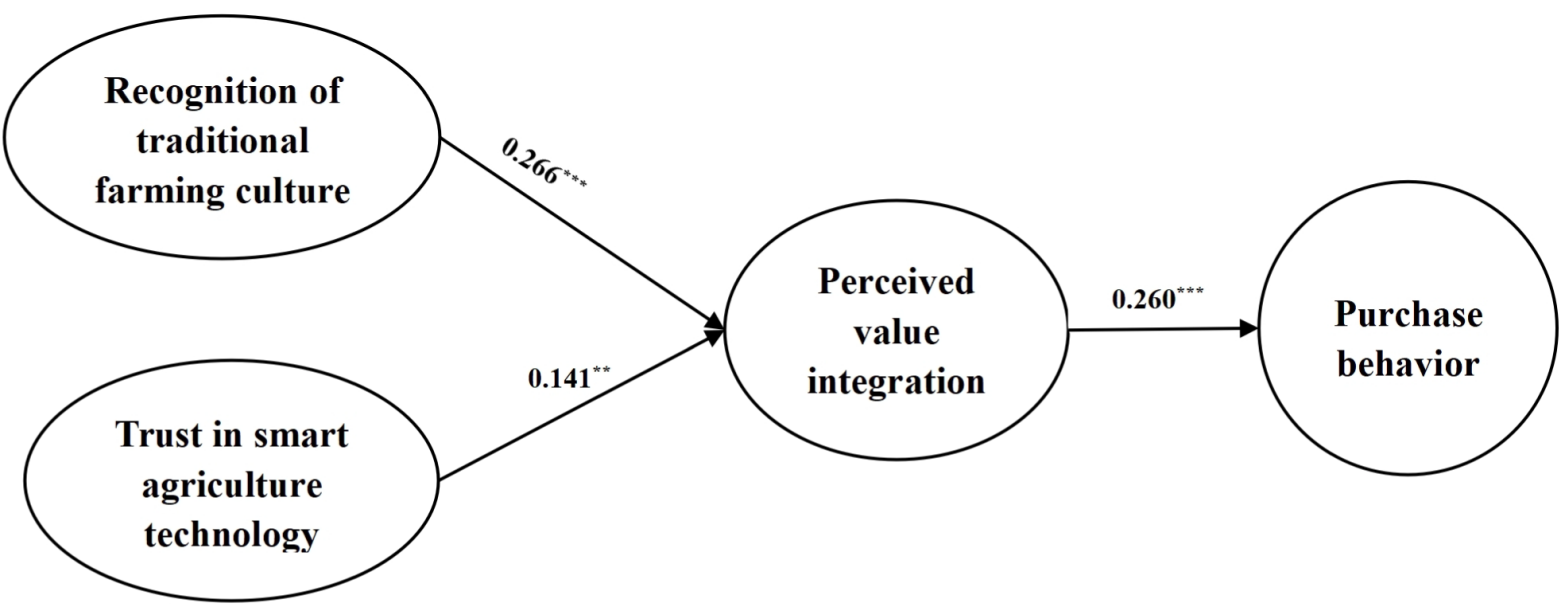
Standardized path diagram of the structural equation model. **p < 0.01; ***p < 0.001.

To improve the transparency of the empirical analysis, the standardized structural model is presented in Figure 2, and the corresponding standardized path coefficients are reported in Table 4. The results show that RTFC positively affects PVI (*β* = 0.266, *t* = 6.865, *p* < 0.001), and TSAT also has a positive effect on PVI (*β* = 0.141, *t* = 2.954, *p* = 0.003). In addition, PVI positively affects PB (*β* = 0.260, *t* = 5.578, *p* < 0.001). These findings support the direct relationships proposed in the structural model.

**Table 4:** Standardized path coefficients and significance levels of the structural model.

Path	*β*	S.E.	*t*-value	*p*-value
RTFC → PVI	0.266	0.039	6.865	<0.001
TSAT → PVI	0.141	0.048	2.954	0.003
PVI → PB	0.260	0.047	5.578	<0.001

The RTFC–TSAT matching effect and the moderating effect of TG were examined separately in the subsequent analyses. Therefore, Figure 2 and Table 4 focus on the main direct paths in the SEM framework.

#### 4.3.2 Testing the nonlinear effects of RTFC–TSAT matching on PVI

Response surface analysis (RSA) with polynomial regression (56) was used to explore how congruence between RTFC and TSAT influences PVI. All predictors were mean-centered, and the distribution of congruence patterns was balanced across groups (RTFC > TSAT: 31.44%; RTFC < TSAT: 29.16%; RTFC = TSAT: 39.40%).

The results indicate that along the line of congruence (LOC: TSAT = RTFC) (Table 5), the slope was significantly positive (*a*_1_ = 0.444, *p* < 0.01), supporting H1a by showing that high and aligned RTFC and TSAT jointly enhance PVI. The curvature was nonsignificant (*a*_2_ = −0.021, *p* = 0.754), indicating no diminishing return. Along the line of incongruence (LOIC: TSAT = -RTFC), the slope was nonsignificant (*a*_3_ = −0.161, *p* = 0.319), whereas the curvature was significantly negative (*a*_4_ = −0.223, *p* < 0.05), signifying that misalignment undermines PVI. The principal axis showed close alignment with the LOC (*a*_5_ = 0.099, *p* = 0.15), which corroborates H1b by confirming that congruence is more beneficial than asymmetry.

**Figure 3:**
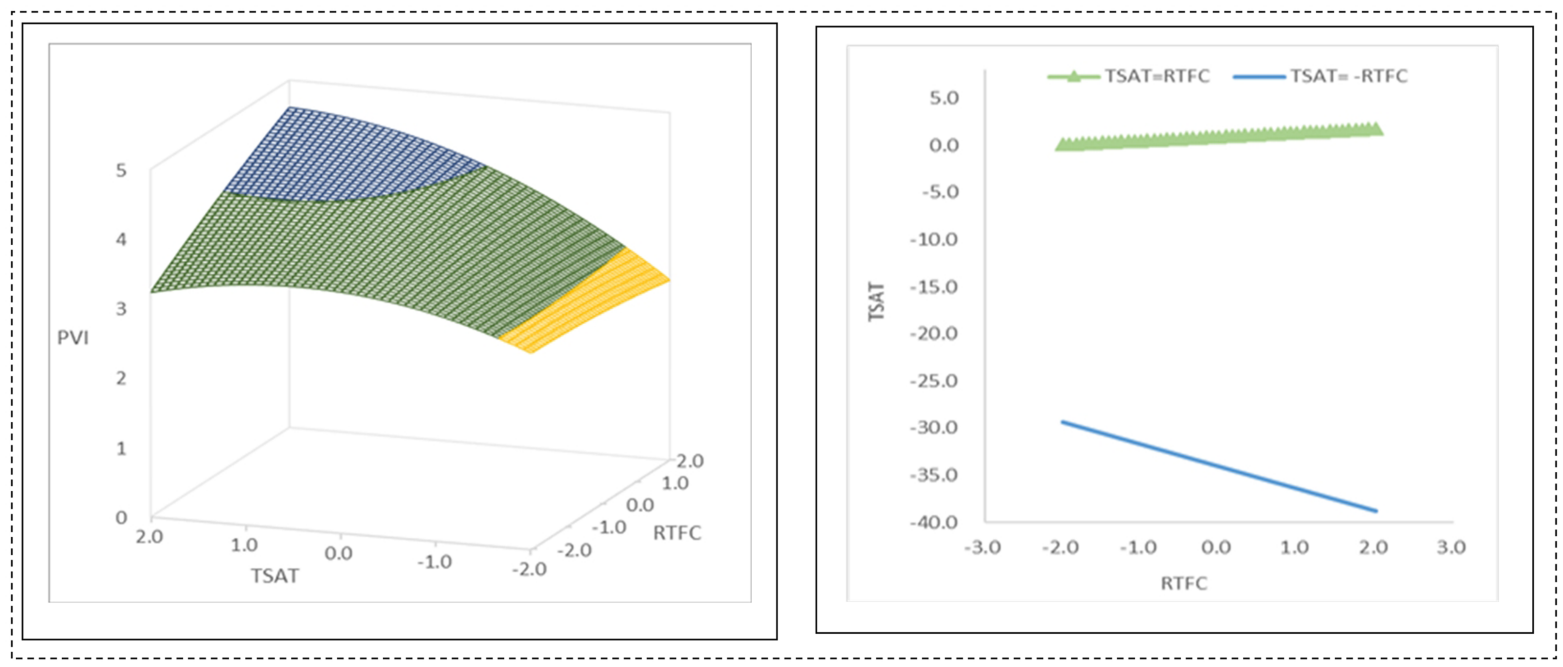
Response surface plot for PVI as shaped by RTFC-TSAT congruence/incongruence.

**Figure 4:**
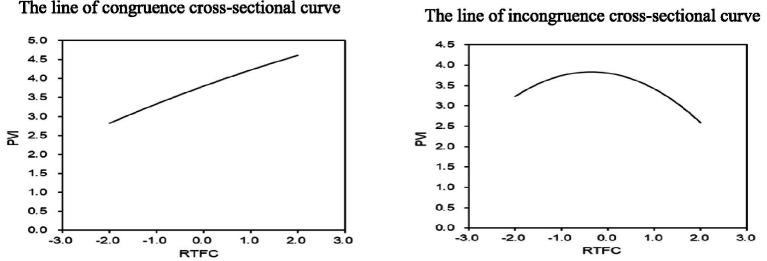
Line of the congruence/incongruence cross-sectional curve graph.

The three-dimensional RSA plots (Figures 3, 4) visually illustrate this pattern: PVI reaches its highest levels when both RTFC and TSAT are high and congruent, while misalignment yields a clear decline.

**Table 5:** Response surface analysis results.

Variable	*β*	S.E
Independent variable		
RTFC (*b*_1_)	0.142	0.099
TSAT (*b*_2_)	0.303^*^	0.121
RTFC^2^ (*b*_3_)	−0.012	0.037
RTFC × TSAT (*b*_4_)	0.101	0.063
TSAT^2^ (*b*_5_)	−0.110^*^	0.054
Surface testing		
The line of congruence (LOC: TSAT = RTFC)		
*a*_1_: Slope (*b*_1_ + *b*_2_)	0.444^**^	0.151
*a*_2_: Curvature (*b*_3_ + *b*_4_ + *b*_5_)	−0.021	0.068
The line of incongruence (LOIC: TSAT = −RTFC)		
*a*_3_: Slope (*b*_1_ − *b*_2_)	−0.161	0.161
*a*_4_: Curvature (*b*_3_ − *b*_4_ + *b*_5_)	−0.223^*^	0.104
*F*	16.442^***^	

*p < 0.05, **p < 0.01, ***p < 0.001; *N* = 703.

#### 4.3.3 Empirical verification of the mediating effect of PVI

To examine whether perceived value integration (PVI) mediates the relationship between congruence in recognition of traditional farming culture (RTFC) and trust in smart agricultural technology (TSAT), and consumer purchase behavior (PB), the block variable (BV) approach proposed by Edwards and Cable (32) was applied. Five polynomial terms—RTFC, TSAT, RTFC^2^, RTFC × TSAT, and TSAT^2^—were weighted by their respective regression coefficients and merged into a composite BV that captures the overall alignment effect between RTFC and TSAT.

Using this BV as the independent variable, mediation testing was conducted with Model 4 of Hayes’ PROCESS macro (SPSS 29.0). As shown in Table 6, BV significantly predicted PVI (*β* = 0.229, *p* < 0.001), indicating that greater congruence enhances consumers’ perceived integration of cultural and technological values. BV also exerted a direct effect on PB (*β* = 0.364, *p* < 0.001), while PVI itself showed a significant positive influence on PB (*β* = 0.303, *p* < 0.001). These findings suggest that PVI serves as an intermediary pathway linking culture-technology congruence to consumer behavior.

**Table 6:** Regression results of the mediation model.

Variable	Model 1		Model 2		Model 3	
	PB		PVI		PB	
	*β*	S.E	*β*	S.E	*β*	S.E
Control variable						
Gender	0.095	0.070	0.073	0.071	0.075	0.068
Age	0.085	0.047	−0.102^*^	0.047	0.112^*^	0.045
Education	−0.089	0.057	−0.455^***^	0.057	0.032	0.057
Income	0.111^**^	0.034	0.053	0.035	0.097^**^	0.033
Independent variable						
BV	0.364^***^	0.035	0.229^***^	0.035	0.303^***^	0.035
PVI					0.267^***^	0.036
*R* ^2^	0.170		0.148		0.230	
*F*	28.468^***^		21.136^***^		34.693^***^	

**p < 0.01, ***p < 0.001; *N* = 703.

To further validate this mediation, a bootstrapping analysis with 5,000 resamples was conducted using the product-of-coefficients method. The indirect effect was 0.061, with a 95% confidence interval [0.034, 0.093] that excluded zero (Table 7), providing robust support for the mediating role of PVI and confirming H2.

**Table 7:** Mediation effect testing.

Path	Effects	S.E	Bootstrap 95% CIs	
			LLCI	ULCI
Total effect (BV → PB)	0.364	0.035	0.295	0.432
Direct effect (BV → PB)	0.303	0.035	0.235	0.371
Indirect effect (BV → PB)	0.061	0.015	0.034	0.093

#### 4.3.4 Empirical test of the moderating effect of TG

The moderating effect of trust in government (TG) was evaluated using the PROCESS macro (Model 58). As reported in Table 8, the interaction term BV × TG was negative and statistically significant (*β* = −0.073, *p* < 0.05; 95% CI [−0.129, −0.016]), indicating that TG weakens the positive influence of cultural–technological congruence on perceived value integration (PVI). This result is opposite to the hypothesized positive moderating effect. Therefore, H3a is rejected.

**Table 8:** Results of the moderation analysis.

Variable	Model 4 (PVI)			Model 5 (PB)		
	*β* (S. E)	Bootstrap 95% CIs		*β* (S. E)	Bootstrap 95% CIs	
		LLCI	ULCI		LLCI	ULCI
BV	0.177^***^ (0.037)	0.104	0.251	0.257^***^ (0.035)	0.187	0.326
PVI				0.249^***^ (0.036)	0.178	0.319
TG	0.076 (0.040)	−0.002	0.153	0.184^***^ (0.036)	0.113	0.256
BV × TG	−0.073^*^ (0.029)	−0.129	−0.016			
PVI × TG				0.018 (0.029)	−0.038	0.074
*R* ^2^	0.167			0.258		
*F*	19.869^***^			30.227^***^		

*p < 0.05, ***p < 0.001; *N* = 703.

By contrast, the interaction term PVI × TG was not significant (*β* = 0.018, *p* = 0.524; 95% CI [−0.038, 0.074]), indicating that TG does not significantly moderate the relationship between PVI and purchase behavior (PB). Therefore, H3b is not supported.

**Figure 5:**
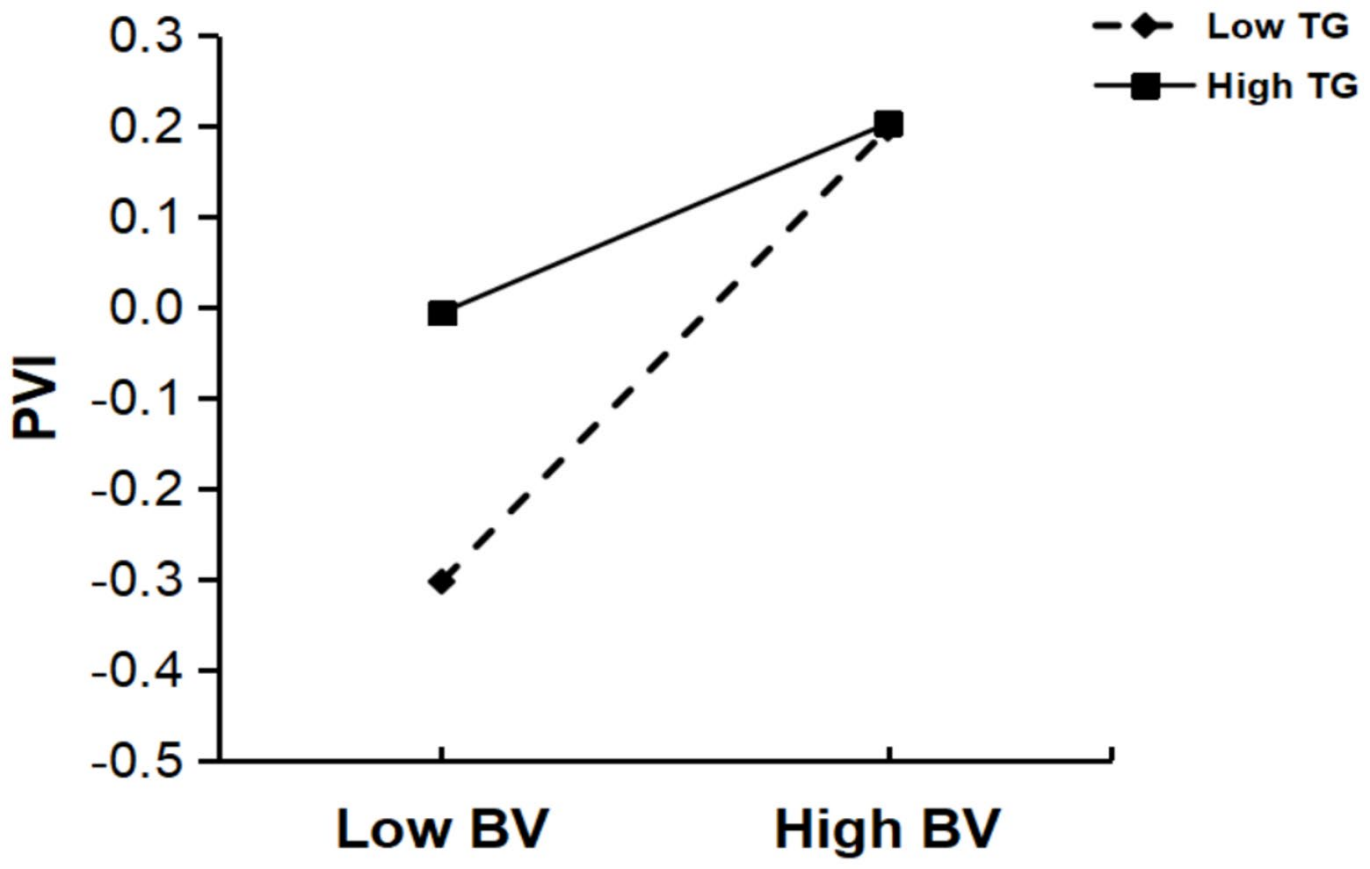
Moderating effect of TG on the relationship between BV and PVI.

A simple slope test (62) further showed that the positive effect of BV on PVI was stronger at low levels of TG (−1 SD) than at high levels of TG (+1 SD). As illustrated in Figure 5, higher TG reduces consumers’ sensitivity to culture–technology fit, thereby weakening its contribution to evaluations of smart agricultural products.

## 5 Discussion

### 5.1 Main findings

This study examined how recognition of traditional farming culture (RTFC) and trust in smart agricultural technology (TSAT) jointly shape consumer purchase behavior (PB) in the context of smart agricultural products. The results show that both RTFC and TSAT positively influence perceived value integration (PVI), which in turn promotes PB. These findings suggest that consumers are more willing to purchase smart agricultural products when they can integrate the symbolic value of traditional farming culture with the functional and trust-related value of smart technology.

The findings further suggest that consumers do not evaluate tradition and technology as entirely separate attributes. Rather, what matters is whether these two value sources can be combined into a coherent overall perception. In the context of smart agriculture, purchase behavior is more likely when consumers perceive that technological advancement does not undermine traditional agricultural meanings, but instead complements or reinforces them.

A particularly noteworthy finding concerns the moderating role of trust in government (TG). Although H3a predicted a positive moderating effect, the empirical results showed a negative and significant coefficient. Therefore, H3a is rejected. This unexpected result is theoretically meaningful. One possible explanation is that when consumers have high trust in government, they may rely more on external institutional assurance—such as regulation, certification, and official supervision—than on their own evaluation of whether traditional farming culture and smart technology are well aligned. In this case, government trust may reduce consumers’ reliance on culture–technology compatibility as a basis for forming PVI, thereby weakening the positive effect of RTFC–TSAT matching on PVI.

This finding adds nuance to the role of institutional trust in food and agricultural consumption. Existing literature often suggests that institutional trust reduces uncertainty and facilitates the acceptance of new products or technologies. However, our results indicate that in culturally embedded consumption settings, strong institutional trust does not necessarily strengthen consumers’ internal integration of cultural and technological values. Instead, it may lessen the extent to which consumers actively reconcile these value dimensions on their own. This pattern may be particularly relevant in the Chinese context, where government regulation and official certification often serve as strong trust signals in food-related decisions.

Overall, the unsupported result for H3a should not be viewed merely as a deviation from the original hypothesis, but as evidence of an important boundary condition: the positive effect of culture–technology fit on perceived value integration may become weaker when external institutional trust becomes a dominant source of reassurance.

### 5.2 Theoretical implications

This study makes several theoretical contributions. First, it contributes to the literature on smart agricultural consumption by identifying perceived value integration (PVI) as a key mechanism linking culture–technology fit to purchase behavior. Previous studies have often focused on cultural preference or technology acceptance separately. In contrast, this study shows that consumer responses to smart agricultural products depend not only on whether tradition is valued or technology is trusted, but also on whether these two dimensions can be integrated into a unified value perception.

Second, this study enriches the literature on culture–technology relationships in agri-food consumption. By jointly considering RTFC and TSAT, it moves beyond a simple “tradition versus modernity” perspective and highlights the possibility that traditional cultural values and technological innovation can coexist and reinforce one another. This perspective is especially relevant in the smart agriculture era, where technological innovation increasingly enters domains with strong cultural and symbolic meanings.

Third, the finding that TG negatively moderates the effect of RTFC–TSAT matching on PVI extends the trust literature by revealing a more complex role for institutional trust. Prior research has mainly emphasized the enabling role of trust in reducing uncertainty and encouraging adoption. In contrast, our findings suggest that institutional trust may also reduce consumers’ reliance on their own value integration process. This result helps clarify the boundary conditions of trust theory in the context of culturally embedded technological products.

### 5.3 Practical implications

The findings provide several practical implications for firms producing and marketing smart agricultural products.

First, firms should not rely solely on technological sophistication as the primary selling point. Although digital traceability, intelligent monitoring, and data-based quality control are important, consumers may respond more positively when such technologies are framed as supporting rather than replacing traditional farming values. Marketing communication should therefore emphasize continuity between traditional agricultural wisdom and technological innovation, including authenticity, ecological harmony, craftsmanship, and respect for local agricultural heritage.

Second, firms should strengthen consumers’ trust in smart agricultural technology through transparent and understandable information. Practical measures may include product traceability systems, visible production records, intelligent monitoring data, third-party certification, and clear explanations of how smart technologies improve food quality, safety, and consistency. These mechanisms can help reduce uncertainty and enhance the perceived compatibility between culture and technology.

Third, because PVI plays an important role in shaping purchase behavior, managers should pay attention to how consumers interpret the overall meaning of smart agricultural products. Product narratives, labels, packaging, and promotional materials should communicate that smart agriculture is not a break from tradition, but a way of preserving traditional values under modern production conditions.

### 5.4 Policy implications

This study also has several policy implications. First, policymakers should continue to improve the institutional environment surrounding smart agricultural products, including regulatory transparency, traceability standards, quality certification systems, and public information disclosure. These efforts can strengthen baseline consumer trust in the market and support the healthy development of the smart agriculture sector.

Second, public policy should not focus only on regulatory assurance, but also on cultural communication. Because consumers’ purchase decisions are influenced by whether they perceive technology as compatible with traditional agricultural values, government agencies and public institutions should promote educational and communication initiatives that explain how smart agriculture can enhance food safety and efficiency while preserving ecological values and agricultural heritage.

Third, demonstration projects, public campaigns, and region-specific agricultural branding may help consumers better understand the connection between tradition and technology. Such initiatives may be especially useful in markets where consumers remain uncertain about whether smart agricultural products are trustworthy, safe, and culturally acceptable.

## 6 Conclusion

This study explored how recognition of traditional farming culture and trust in smart agricultural technology jointly influence consumer purchase behavior in the smart agriculture era. The results indicate that RTFC and TSAT both positively contribute to perceived value integration, which in turn promotes purchase behavior toward smart agricultural products. The study also finds that trust in government negatively moderates the relationship between RTFC–TSAT matching and PVI, suggesting that strong institutional trust may weaken consumers’ reliance on culture–technology compatibility when forming value perceptions.

These findings highlight the importance of value integration in explaining consumer responses to smart agricultural products and provide a more nuanced understanding of the role of institutional trust in culturally embedded technology adoption.

This study has several limitations. First, the cross-sectional design limits strong causal inference. Future research could adopt longitudinal or experimental approaches to better capture the dynamic formation of value integration and purchase behavior. Second, the study focuses on the Chinese context, where traditional agricultural culture and government trust may operate in distinctive ways; future studies should test the proposed framework in other cultural settings to examine its generalizability. Third, additional factors such as product category, consumer knowledge, perceived risk, and regional differences may further influence the relationship between culture–technology fit and purchase behavior and should be incorporated into future research.

## Data Availability

The datasets presented in this study can be found in online repositories. The names of the repository/repositories and accession number(s) can be found in the article/supplementary material.

## Footnotes

1
www.credamo.com

2
www.stats.gov.cn

## Appendix. Measurement items

**Table:**

Construct and items (references)	Loadings
Recognition of traditional farming culture [adapted from (50, 51)]	
1. I believe traditional agricultural practices carry important cultural and historical values.	0.771
I highly respect the wisdom embedded in traditional farming methods.	0.637
3. Even in today’s tech-driven world, valuable traditional practices should be preserved.	0.676
4. Traditional agriculture reflects the harmonious relationship between humans and nature.	0.758
Trust in smart agriculture technology [adapted from (41, 52, 53)]	
1. I believe smart agriculture technologies can improve food safety effectively.	0.782
2. I believe the traceability information provided by smart agricultural products is reliable.	0.731
3. I trust the accuracy and security of the data collection and monitoring systems used in smart agriculture.	0.741
4. I believe the application of smart agricultural technologies is scientific, controllable, and beneficial.	0.734
Perceived value integration [adapted from (37, 54)]	
1. I believe that traditional agricultural values (e.g., nature, craftsmanship) and modern smart agriculture (e.g., precision, efficiency) can coexist.	0.777
2. For smart agricultural products, I can appreciate both the convenience brought by technology and the cultural or natural values embedded within them.	0.745
3. In my view, eco-friendly and natural consumption philosophies are complementary to the application of intelligent technologies.	0.796
Trust in government [adapted from (24, 43)]	
1. I believe the government is honest and responsible in promoting smart agriculture.	0.813
2. I believe the government has the capability to formulate and implement effective policies to support smart agricultural development.	0.719
3. I trust that the safety standards and regulations established by the government for smart agricultural products are rigorous and effective.	0.755
4. Government actions in smart agriculture enhance my confidence in these products.	0.754
Purchase behavior [adapted from (55)]	
1. I frequently purchase smart agricultural products.	0.867
2. Even when traditional agricultural products are on promotion, I still prefer to choose smart agricultural products.	0.837
3. I am willing to pay a higher price for smart agricultural products compared to traditional ones.	0.879
